# Doctors and Lodges: The Commission's Finding

**Published:** 1918-09-14

**Authors:** 


### AUSTRALIAN NOTES.

### Doctors and Lodges: The Commission's Finding.

Judge Wasley, sitting as a Commission on the dispute between the B.M.A. and the Association of Friendly Societies of Victoria, issued the major portion of his finding on June 21, after a week's consideration of the evidence given during the previous five or six weeks. The finding states that from 1878 to 1916 the rates of medical pay from Friendly Societies had remained practically unchanged. At a conference in August 1913 the B.M.A. set before the Friendly Societies their arguments for an increase of pay. A second conference was held in July 1914, and at a meeting of a sub-committee from each side held on August 8, 1914, four days after the outbreak of war, a resolution was carried that the consideration of the matter be postponed to some future date. The Judge states that he feels certain that some, if not all, of the Friendly Societies' representatives believed that "some future date" meant that the dispute was to be allowed to lie dormant until after the war—neither side dreaming that the war would last four years.

Judge Wasley stated that, coming to the claim for increased payment, he had taken into account the facts that when a doctor obtains a lodge appointment payment for his services is guaranteed without effort on his part, and that a lodge appointment gives the doctor opportunities he would not otherwise have of working up a remunerative private practice.

On the other hand, wages of adult male workers in Australia had increased on the average by 55 per cent. since 1891. Also, doctors as well as wage-earners were affected by the increased cost of commodities. The rate demanded, 20s. per annum for town members, he thought not unfair. In the past, country rates had been 25 per cent. higher than town rates, so applying this principle he suggested 25s. per member per annum as a fair charge for country members. (The doctors had asked for 26s.) He considered that portion of this increase should not be payable until after the war. He would suggest that the rate reduction be 3s. in each case. Thus, during war-time, town rate to be 17s., country rate 22s. After war, town rate 20s., country rate 25s.

Income limitation was a more difficult matter. Judge Wasley suggests these limitations to apply only to new members and to have no retrospective effect. As Judge Wasley sat only as a Royal Commission and not as a statutory official there is no power anywhere to enforce the acceptance of these recommendations on either side of the two parties to the dispute. The doctors are understood to be pleased with the finding; the Friendly Societies not wholly displeased. The remainder of the report is awaited with curiosity, if not anxiety, as it is expected to deal with the knotty question of the lately established medical institutes.

